# Behavioural and physiological impacts of low salinity on the sea urchin *Echinus esculentus*

**DOI:** 10.1242/jeb.246707

**Published:** 2024-01-16

**Authors:** Nicholas J. Barrett, Elizabeth M. Harper, Kim S. Last, Helena C. Reinardy, Lloyd S. Peck

**Affiliations:** ^1^ British Antarctic Survey, Natural Environment Research Council, Cambridge CB3 0ET, UK; ^2^ Department of Earth Sciences, University of Cambridge, Cambridge CB2 3EQ, UK; ^3^ The Scottish Association for Marine Science, Oban PA37 1QA, UK; ^4^ Department of Arctic Technology, The University Centre in Svalbard, N-9171 Longyearbyen, Norway

**Keywords:** Osmotic stress, Echinoderm, Acclimation, Coastal freshening, Climate change, Phenotypic plasticity

## Abstract

Reduced seawater salinity as a result of freshwater input can exert a major influence on the ecophysiology of benthic marine invertebrates, such as echinoderms. While numerous experimental studies have explored the physiological and behavioural effects of short-term, acute exposure to low salinity in echinoids, surprisingly few have investigated the consequences of chronic exposure, or compared the two. In this study, the European sea urchin, *Echinus esculentus*, was exposed to low salinity over the short term (11‰, 16‰, 21‰, 26‰ and 31‰ for 24 h) and longer term (21, 26 and 31‰ for 25 days). Over the short term, oxygen consumption, activity coefficient and coelomic fluid osmolality were directly correlated with reduced salinity, with 100% survival at ≥21‰ and 0% at ≤16‰. Over the longer term at 21‰ (25 days), oxygen consumption was significantly higher, feeding was significantly reduced and activity coefficient values were significantly lower than at control salinity (31‰). At 26‰, all metrics were comparable to the control by the end of the experiment, suggesting acclimation. Furthermore, beneficial functional resistance (righting ability and metabolic capacity) to acute low salinity was observed at 26‰. Osmolality values were slightly hyperosmotic to the external seawater at all acclimation salinities, while coelomocyte composition and concentration were unaffected by chronic low salinity. Overall, *E. esculentus* demonstrate phenotypic plasticity that enables acclimation to reduced salinity around 26‰; however, 21‰ represents a lower acclimation threshold, potentially limiting its distribution in coastal areas prone to high freshwater input.

## Teaser

**Highlighted Article:** The sea urchin *Echinus esculentus* demonstrates acclimation abilities to reduced salinity conditions of 26‰; however, salinities around 21‰ are expected to act as a threshold, limiting its survival and distribution in the context of climate change-induced coastal freshening.

## INTRODUCTION

Salinity is one of the main abiotic factors impacting the physiology and ecology of marine and brackish water organisms (Kinne, 1964) and is key to determining species distribution, reproductive success, growth and survival (Gunter, 1961; McNamara and Faria, 2012). Freshwater input in coastal and estuarine environments can reduce seawater salinity (coastal freshening) and exert hypo-osmotic stress on marine organisms by increasing the cost of osmoregulation (Sokolova et al., 2012) and even lead to cellular damage (Jäckle et al., 2001; Lee et al., 2022). Climate change is predicted to cause increased coastal freshening in many areas, primarily driven by increased rainfall and glacial melting, thus contributing to increases in freshwater land runoff (Jacobs et al., 2002; IPCC, 2022). Benthic marine invertebrates are considered particularly vulnerable to coastal freshening (Moon et al., 2015; Vuorinen et al., 2015) because of their characteristically limited mobility (Snelgrove, 1999), making it a challenge to evade rapid changes in salinity.

Members of the phylum Echinodermata, as with the majority of marine invertebrates, are osmoconformers and are poorly equipped to regulate extracellular body fluid concentrations in response to changes in external salinity (Gilles, 1987; Hyman, 1955; Stickle and Diehl, 1987). However, many echinoderm species are frequently found in the intertidal and even brackish environments, demonstrating a degree of euryhalinity (Binyon, 1972; Russell, 2013; Stickle and Diehl, 1987). For tolerant species, short-term abrupt reductions in salinity appear to be countered by the establishment of transient ionic and osmotic gradients between the internal coelomic fluid and the external seawater, suggesting a degree of epithelial regulation (Freire et al., 2011; Santos et al., 2013). This is considered a buffering mechanism to protect internal cellular tissue from swelling on encountering fresher water – for example, over a tidal cycle – enabling time for cellular volume regulation (Vidolin et al., 2007). Cell volume regulation in osmoconformers involves adjusting concentrations of compatible organic and inorganic ions, known as osmolytes, to balance cell osmotic pressure (Gilles and Delpire, 1997; Podbielski et al., 2022). With abrupt changes in salinity, intracellular inorganic osmolytes can be rapidly released (e.g. K^−^ and Cl^−^), followed later by organic osmolytes (e.g. taurine) (Smith and Pierce, 1987), preventing structural cell damage. However, with chronic exposure to reduced salinity, organic osmolyte composition and concentration are gradually modified, favouring selection for osmolytes with the best osmoprotecting qualities (Somero et al., 2017). This may involve cellular reorganisation with modifications to membrane-bound transporters (Gilles, 1987; Meng et al., 2013) and even cell cytoskeleton structural changes (Barrett et al., 2022; Pedersen et al., 2001). These energy-dependent, long-term physiological adjustments occur during the process of acclimation to reduced salinity, which is considered to take between 1 and 4 weeks in temperate invertebrates, i.e. bivalve and gastropod molluscs, amphipods and jellyfish (Khlebovich, 2017). The extent to which organisms can acclimate to changing environmental conditions is considered key to defining their future success under climate change scenarios (Peck, 2011; Somero, 2012). Experimental approaches investigating salinity tolerance in echinoids have mainly focused on behavioural and metabolic responses to short-term acute exposure, and only occasionally longer term acclimation (see Binyon, 1972; Stickle and Diehl, 1987; Russell, 2013). Rarely have short- and long-term tolerance been compared; indeed, reviews often compare the lower tolerance limits of different species without distinguishing the duration of the experiment (e.g. Russell, 2013).

The European sea urchin (hereafter ‘urchin’), *Echinus esculentus* Linnaeus 1758, is a widespread member of the benthic north-east Atlantic temperate ecosystem, commonly associated with the giant kelp forests of *Laminaria hyperborea* (Comely and Ansell, 1988). Although occasionally exposed at low tide, *E. esculentus* is most abundant in the sub-littoral zone from ∼15 to 40 m, where densities of 4 m^−2^ have been recorded (Comely and Ansell, 1988), and is rarely found at depths >40 m (Comely and Ansell, 1988; Mortensen, 1927). *Echinus esculentus* plays a prominent role in the ecology of the sublittoral zone (Forster, 1959), exerting control on the distribution of *Laminaria* spp. and numerous epiphytic macroalgae through grazing pressures (Jones and Kain, 1967). Indeed, their grazing impact may be detrimental to kelp forest recovery in a dysfunctional ecosystem (Bekkby et al., 2015). However, *E. esculentus* is listed on the IUCN Red List as ‘near threatened’, citing severe population fragmentation and declines in numbers of mature individuals (www.iucnredlist.org/species/7011/12821364, accessed 29 August 2023). As a subtidal inhabitant, they are less likely to experience dramatic reductions in surface salinity than urchins more commonly found in the intertidal (e.g. *Psammechinus miliaris*; Kelly and Cook, 2001) and therefore may be predicted to have lower tolerance to osmotic stress. Previous investigations of *E. esculentus* have been concerned with the impacts of commercial exploitation around the British Isles for both consumption of urchin roe and the collection of urchin tests for ornamental purposes (Comely and Ansell, 1988, 1989; Kelly et al., 2001). However, there is little to no knowledge on the salinity tolerance of *E. esculentus* and its capacity to acclimatise to a fresher coastal environment. In this study, experiments were conducted on populations of *E. esculentus* originally collected from Loch Linnhe, west coast of Scotland. Salinity in Scottish coastal waters has been dramatically decreasing since 2014 (Dye et al., 2020). Winter rainfall on the west coast of Scotland is expected to rise on average by 19% under a high emission scenario by 2080 (Adaptation Scotland, www.adaptationscotland.org.uk/why-adapt/climate-trends-and-projections, accessed 29 August 2023). Understanding the degree to which *E. esculentus* can tolerate reductions in salinity may allow predictions about its future distribution under increased coastal freshening (i.e. migration to deeper, higher salinity water), and how this may impact local ecosystem functioning (i.e. by reducing macroalgal grazing pressure in shallower areas).

Through investigating physiological and behavioural responses to reduced salinity over different time frames, this study aims to decouple the effects of acclimation from short-term, immediate responses to osmotic shock. Three hypotheses were addressed: (1) chronic exposure to salinity levels at the lowest acutely tolerated level will have a detrimental impact on routine physiological functions (e.g. respiration, feeding and righting abilities) and, in addition, may impact innate immune cell (coelomocytes) composition and concentration, as has previously been shown (Honorato et al., 2017); (2) urchins exposed to a mid-level salinity within their tolerance range will acclimate, evidenced by a return of physiological functions to levels that are indistinguishable from those at ambient salinity; and (3) if acclimation is achieved, future hypo-osmotic stress will be reduced as a result of the cellular adjustments established during the acclimation process.

## MATERIALS AND METHODS

### Experimental animals

Individual *E. esculentus* (*n*=140) with a test diameter of 21–57 mm were selected from stock originally collected from Loch Linnhe, then maintained in flow-through aquaria. The aquarium utilises filtered natural seawater directly from the waters surrounding the Dunstaffnage Peninsula at the Scottish Association of Marine Science (SAMS) aquarium, Scotland. Urchins were transferred to a temperature-controlled room (13.7±0.3°C, mean±s.e.m., *n*=7) and held in replicate 50 l closed-circuit aquaria (maximum *n*=20 per tank) for 7 days prior to each experimental procedure to allow a period of habituation after handling and changing aquaria. Animals were not fed over this period to ensure that when feeding trials commenced, animals were at comparable points in their digestive cycle. Each tank was fitted with a nano protein skimmer (REEF-Skim Nano 100AC, TMC Ltd) and bio-filter (ZB-150, Ziss) with an attached airline. Water was changed daily using UV-sterilised filtered seawater which had a mean (±s.e.m.) salinity of 31±0.05‰ (*n*=7). A photoperiod of 16 h light and 8 h dark was maintained throughout all experiments to represent local summer photoperiods. Urchins were maintained and handled in accordance with UK animal welfare regulations.

### Experiment 1: acute salinity tolerance

Five salinity treatments were used to assess urchin salinity tolerance to an acute challenge over a 24 h period: 31‰ (control), 26‰, 21‰, 16‰ and 11‰. Because of logistical constraints, each treatment was conducted in a single 50 l tank with a set-up as described above, with tank water averaging 13.75±0.17°C (mean±s.e.m., *n*=10). The impact of tank effects was considered minimal given the short duration of the experiment (24 h) in clean water, which was monitored for temperature, salinity and water chemistry, while each urchin was isolated in a floating tray. In addition, the impact of abrupt reductions in salinity on urchin functions was considered to be substantially greater than any potential variations due to tank effects, and therefore each urchin was considered a biological replicate. Reverse osmosis water was mixed with filtered seawater until experimental salinity levels were achieved. Salinity was measured using a conductivity probe (CDC40101, Hach). Ten urchins were selected at random for each experimental salinity treatment (*n*=50 in total). For each treatment, oxygen consumption, righting ability, coelomic fluid osmolality and mortality were assessed. After 24 h, tank water was immediately replaced with ambient seawater (31‰).

### Experiment 2: low salinity acclimation

Three salinity treatments were selected to assess acclimation over a 25 day period based on salinity data from sites of wild collection (e.g. minimum surface salinity was 28‰ and 31‰ in May, and 15‰ and 23‰ in October 2011 and 2012, respectively) (Rabe and Hindson, 2017) and the results of the acute trial: control (31‰), medium (26‰) and low (21‰) salinity. For each salinity treatment, 30 urchins were randomly distributed between three, 50 l tanks (set up as previously described), with a total of 10 urchins per tank (total *n*=90 for the acclimation experiment). This design allowed an evaluation of tank effect variation within salinity treatments to assess whether each individual urchin could be considered a biological replicate.

After a 7 day habituation period, the medium and low salinity tanks were diluted by adding reverse osmosis water to each tank and adjusting for tank volume in a stepwise manner, reducing salinity by ∼2‰ per day until experimental treatment levels were reached (as per Shirley and Stickle, 1982a). Within each salinity treatment, 10 urchins were placed in numbered floating trays (split between the three tanks in each treatment in a ratio of 3:3:4) which facilitated repeated measurements for oxygen consumption, feeding and mass. Water quality was assessed twice a week (Saltwater Master Test Kit, API). Ammonium (NH_4_^+^), nitrates (NO_3_^−^) and nitrites (NO_2_^−^) were kept below 0.05, 1 and 0.025 mg l^−1^, respectively, by refreshing tank water at least twice a week by at least 30 l per tank. Tank water temperature was checked daily (13.76±0.03°C, mean±s.e.m., *n*=25) over the experimental period. Tank water pH was monitored twice weekly using a pH probe (Hach) and remained stable over the experimental period (pH 7.93±0.014, mean±s.e.m, *n*=8). On days 1, 5, 9, 17 and 25 after the target experimental salinity was reached (hereafter termed ‘time points’), oxygen consumption, feeding rate (additionally on day 13), righting ability and coelomic fluid osmolality were assessed. Coelomocyte analysis, wet mass change and mortality were assessed on day 25.

### Experiment 3: hypo-osmotic shock of acclimated urchins

At the end of the acclimation experiment (day 25), all urchins in each treatment were transferred to 18‰ salinity to assess the response to an acute osmotic shock for a 6 h duration. A salinity of 18‰ was chosen as this was 2‰ above the lethal salinity identified in the acute experiment and near the predicted LC_50_ value (18.5‰). Oxygen consumption (*n*=8 per treatment), righting ability (*n*=11 per treatment) and coelomic fluid osmolality (*n*=6 per treatment) were measured. All urchins were then transferred back to their prior acclimated salinity and assessed for mortality over the next 72 h.

### Oxygen consumption

Oxygen consumption was assessed using closed chamber techniques following Suckling et al. (2015) and Morley et al. (2016). Individual urchins (*n*=10 per treatment in experiment 1 and 2, *n*=8 per treatment in experiment 3) were immersed directly into experimental salinity tanks and placed within open respirometry chambers (experiment 1 and 3) or transferred underwater to open respirometry chambers (experiment 2). As handling is likely to affect oxygen consumption as a result of stress, animals were left for 2 h prior to commencing measurements (pre-trial testing in ambient salinity showed that 2 h was sufficient for a return to pre-handling metabolic rate). Once the respirometry chambers were sealed, oxygen concentration was measured using a Fibox-4 fibre optic oxygen sensor (PreSens). After 3 h in the closed chambers, a second measurement was taken; 3 h was sufficient to allow a drop in oxygen levels from full saturation to 90–70%. Three control chambers without animals were measured to account for background oxygen changes, which were used to correct the calculated animal oxygen consumption rates. Chamber volume was adjusted for animal volume (obtained by displacement) to produce a ‘respired water volume’ used in calculating oxygen consumption. In experiment 2, oxygen consumption was measured in the same 10 urchins within each treatment at each time point. Oxygen consumption was measured at approximately the same time of day (mid-morning) to minimise any circadian rhythm effects. Each time point measurement was scheduled 4 days after the last feeding assessment to minimise the impact of raised metabolism associated with feeding (the specific dynamic action of feeding, SDA). Temperate marine invertebrates typically require 2–5 days to return to basal oxygen consumption levels after feeding (Secor, 2009). Animal wet mass (±0.01 g), volume (±0.01 g) and test diameter (±0.5 mm) were recorded for each urchin. To obtain ash-free dry mass (AFDM; ±0.01 g), all animals were euthanised by freezing at −20°C and then dried in a convection oven at 60°C to constant mass (±0.01 g). Dried urchins were then transferred to a muffle furnace, which was heated to 475°C for 6 h. AFDM was obtained by subtraction and used to estimate the mass of respiring organic tissue in the animal.

### Righting ability

Urchins orientate oral-side down; inverting them elicits a righting response. The time taken for urchins to fully right themselves was recorded and converted to an activity coefficient (AC=1000/righting time in seconds) after Lawrence and Cowell (1996). The maximum time allocated for a righting response was 30 min, which equals a minimum AC value of 0.55 (1000/1800 s). Care was taken when handling urchins to minimise damage to tube feet that were attached to the tank surface, by gently applying pressure and waiting for tube feet to detach. In experiment 1 (*n*=5 per treatment) and experiment 3 (*n*=11 per treatment), AC was assessed after 5 h in the experimental salinity. In experiment 2, AC assessments were performed on 7–10 animals per treatment at each time point.

### Coelomic fluid osmolality

Coelomic fluid samples were taken by sub-lethal extraction to assess osmolality (*n*=5 per treatment in experiment 1 after 24 h; *n*=5 per treatment in experiment 2; *n*=6 per treatment in experiment 3 after 6 h). A syringe with a 21-gauge needle was inserted through the ﻿peristomial membrane at a 45 deg angle away from the oral area and into the coelomic cavity (after Reinardy and Bodnar, 2015). Approximately 1 ml of coelomic fluid was extracted per urchin. Coelomic fluid was centrifuged (800* **g*** for 4 min) in sterile microcentrifuge tubes to separate the coelomocyte pellet from the coelomic fluid supernatant. The supernatant was pipetted into a separate tube and stored at −80°C for later analysis. Triplicate tank water samples were collected at each sample point and a mean seawater osmolality obtained for use in one-way *t*-test comparisons with coelomic fluid. The osmolality of the coelomic fluid and seawater was measured on a Vapro 5600 vapour pressure osmometer (ELITech Group).

### Mortality

After experiments 1 and 3, animals were examined every 24 h (up to 5 days) for signs of life (observed movement of spines and tube feet) and re-tested for their righting response. Mortality rates were assessed on animals that had not been subjected to coelomic fluid extraction. In experiment 2, urchin mortality was monitored daily, with urchins that appeared dead (i.e. inability to right after 24 h and lack of movement in spines and tube feet) removed.

### Feeding rate (experiment 2 only)

Feeding trials began when urchins had reached their experimental salinity and was repeated every 4 days. A diet of brown kelp (*Laminaria* spp.) was offered as it had been previously used to feed urchins at SAMS, is locally abundant and is known to be a main source of food in the wild (Bekkby et al., 2015; Jones and Kain, 1967). A pre-weighed amount of fresh ﻿kelp was offered to each urchin isolated in a tray and left for 24 h (based on initial feeding trials to assess amount). After 24 h, remaining kelp was removed and re-weighed, with the difference taken as the amount eaten. Buoyant mass was selected as a weighing metric (adapted from Jokiel et al., 1978), as this is insensitive to water contained in the fresh porous kelp tissue. For the remaining urchins which were not being assessed for their feeding rate, excess un-weighed quantities of kelp were provided at each feeding point.

### Coelomocyte collection and analysis (experiment 2 only)

Coelomocytes were collected at the last sample point (day 25) from 7 urchins per treatment. Coelomic fluid was collected as previously described, with the addition of using syringes prefilled with 0.5 ml ﻿ice-cold calcium/magnesium-free seawater (460 mmol l^−1^ NaCl, 10 mmol l^−1^ KCl, 7 mmol l^−1^ Na_2_SO_4_, 2.4 mmol l^−1^ NaHCO_3_), containing 30 mmol l^−1^ ethylenediaminetetraacetic acid (EDTA) anticoagulant (pH 7.4) to prevent cell clumping (Liu et al., 2023). A volume of ∼0.5 ml coelomic fluid was extracted per urchin. Cell concentration, differential cell counts (red spherulocytes and clear cells) and cell viability were assessed on a ﻿Neubauer haemocytometer. To assess viable cells, coelomic fluid was mixed with 0.5% Trypan Blue (ratio 1:1) and the number of dead blue cells and clear live cells counted (Liu et al., 2023).

### Wet mass change and percentage AFDM (experiment 2 only)

Wet mass (g) was recorded before and after the 25 day exposure in the isolated urchins (*n*=10 per treatment) with the difference noted, after Podbielski et al. (2022). On both occasions, there was a gap of 7 days between the last feeding point and weighing, to ensure that animals were at similar points in their digestive cycle. The relationship between AFDM (organic mass) and ash mass (inorganic mass) was used to determine changes in organic mass over the trial, expressed as:
(1)

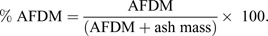


### Statistics

One-way analysis of variance (ANOVA) tests (including Welch's ANOVA for data with unequal variances) were performed at each time point to compare responses between salinity treatments. Two-way ANOVA tests were conducted to compare response variables between treatments, time points and their interactions. Where repeat measurements were conducted on the same animal across different time points, the addition of individual animal as a random factor was included in the model. Within the acclimation experiment, tank effects were assessed on oxygen consumption, feeding rate and righting data utilising a two-way ANOVA with tank and time point as fixed variables, and individual as a random factor when repeat measures were taken. Assumptions of normality and equal variance were formally tested using the Shapiro–Wilk test and Levene's test, respectively. When assumptions of normality were violated, data were either log or square root transformed. With continued violations of normality, non-parametric Kruskal–Wallis tests were implemented. For significant ANOVA/Kruskal–Wallis test results, *post hoc* testing was carried out to determine differences between treatments. Tukey tests were used for normally distributed data, Dunn's test for non-parametric data and Games–Howell tests for data with unequal variance. When assumptions of normality were violated in two-way models, an aligned rank transformation ANOVA (ART ANOVA) was implemented as a non-parametric alternative. One sample *t*-tests were used when comparing coelomic fluid values with mean tank water osmolality. Two sample *t*-tests were used when comparing two group means. All percentage data were arcsine transformed prior to statistical analysis. Statistical tests were considered significant if *P*<0.05. All analyses were carried out in R (version 4.0.5).

## RESULTS

### Experiment 1: acute salinity tolerance

After 24 h, mortality was 100% in the 11‰ and 16‰ salinity treatments. All urchins in the 21‰, 26‰ and control (31‰) treatments survived, showing no significant differences in mean righting time between treatments after 5 days of recovery at 31‰ (Table S1). Mortality was 100% at 16‰ and 0% at 21‰; therefore, if the relationship is linear, the LC_50_ value (salinity which is lethal to 50% of exposed urchins) for *E. esculentus* is estimated to be 18.5‰.

**Fig. 1.**
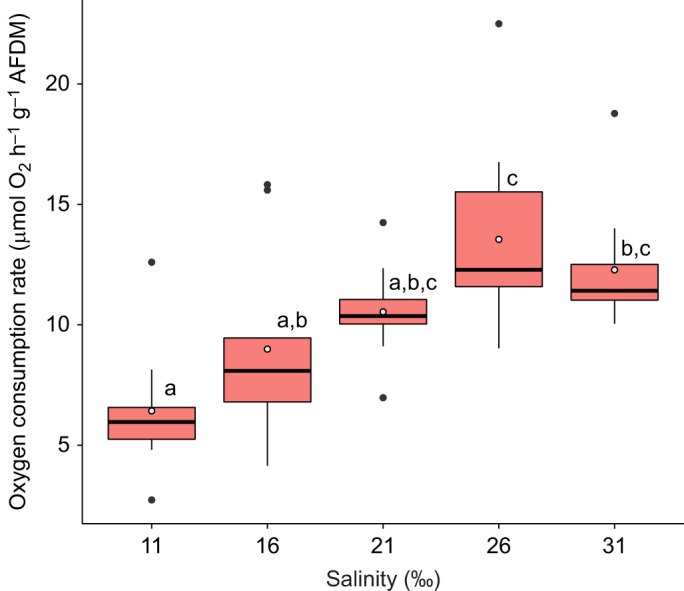
**Oxygen consumption of *Echinus esculentus* 2 h after transfer from ambient salinity (31‰).** Results are shown for Kruskal–Wallis and *post hoc* Dunn’s test, with different letters indicating significant differences (*P*<0.05; *n*=10 biological replicates for each treatment) between treatments. Box plots show medians (black line), upper and lower quartiles, and maximum and minimum (whiskers). Means are represented by open circles and outliers by filled circles. AFDM, ash-free dry mass.

Oxygen consumption differed significantly between treatments (Kruskal–Wallis: χ^2^_4_=22.68, *P*<0.001), with the following treatment comparisons showing significant differences: 11‰ and 26‰, 11‰ and 31‰, 16‰ and 26‰ (Dunn’s test: all *P*<0.03) (Fig. 1; Table S1).

**Fig. 2.**
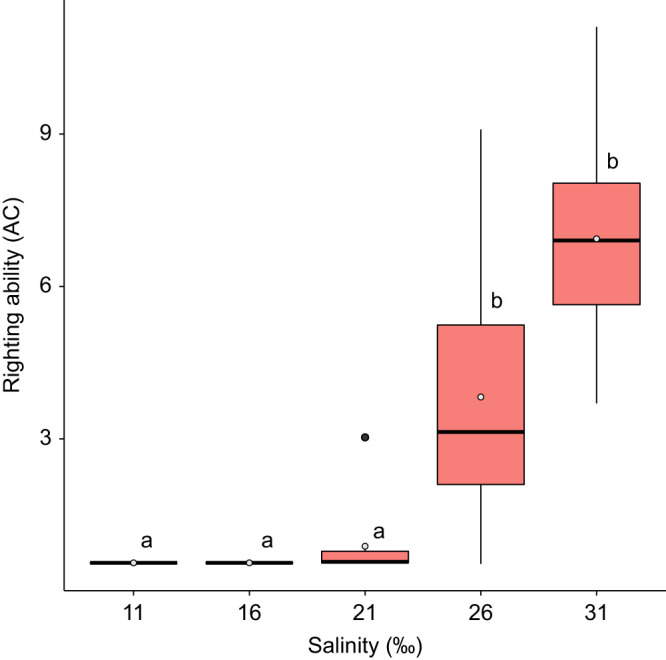
**Righting ability of adult *E. esculentus*** **5** **h after transfer from ambient salinity (31‰).** Righting ability was converted to activity coefficient, calculated as AC=1000/righting time in seconds; a smaller AC value indicates a longer righting time. Results are shown for Kruskal–Wallis and *post hoc* Dunn’s test, with different letters indicating significant differences (*P*<0.05; *n*=10 biological replicates for each treatment) between treatments. Box plots show medians (black line), upper and lower quartiles, and maximum and minimum (whiskers). Means are represented by open circles and outliers by filled circles.

After 5 h of immersion, mean AC values were significantly different between the control treatment and all other salinities (Dunn’s test: all *P*<0.001), except 26‰ (Fig. 2; Table S1). Additionally, AC values at 26‰ significantly differed from those at 21‰, 16‰ and 11‰ (Dunn’s test: all *P*<0.03) (Fig. 2; Table S1). In the 16‰ and 11‰ treatments, no urchins righted within 30 min.

**Fig. 3.**
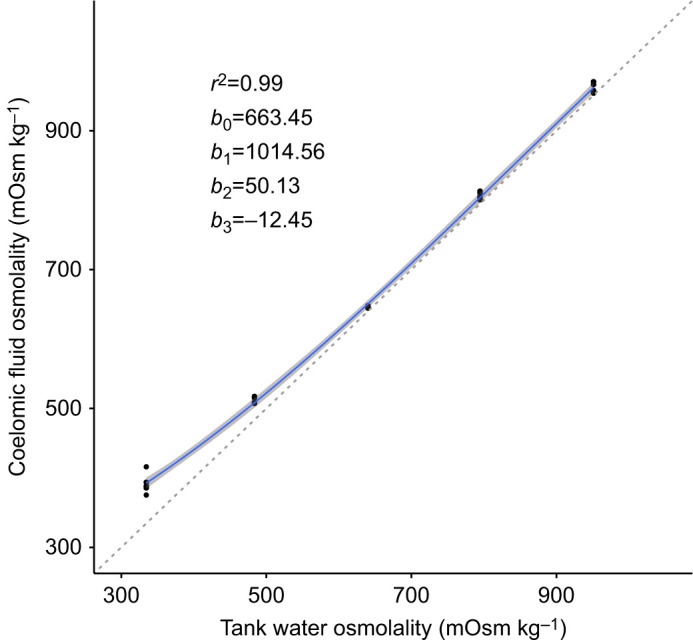
**Coelomic fluid osmolality of adult *E. esculentus* 24 h after transfer from ambient salinity (31‰) versus that of the corresponding tank water.** All coelomic fluid values were significantly different from their corresponding tank water osmolality (one sample *t*-test: all *P*<0.05; *n*=5 biological replicates at each salinity; 31‰, 26‰, 21‰, 16‰ and 11‰ correspond to 951, 794, 640, 484 and 329 mOsm kg^−1^ tank water osmolality, respectively). A regression line (blue line) and the 95% confidence intervals (shaded grey area) were fitted to the data. The iso-osmotic line is represented by the dashed grey line. Linear regression coefficients and *r*^2^ value are included to the left of the regression line.

Mean coelomic fluid osmolality was significantly different between all salinity treatments after 24 h (Welch's ANOVA: *F*_4_=8666, *P*<0.00001; Games–Howell, all *P*<0.0001) (Fig. 3; Table S1). Mean coelomic fluid osmolality was slightly but significantly hyperosmotic to tank seawater in each salinity treatment (one sample *t*-test; all *P*<0.05) (Fig. 3; Table S1).

### Experiment 2: low salinity acclimation

#### Tank effects

There was no significant variation between treatment tanks; therefore, each individual urchin was considered a biological replicate within each treatment (Table S2).

#### Mortality

Over the course of the experiment, there were four mortalities: two in the low salinity treatment (day 10 and day 20), one in the control (day 24) and one accidental death in the medium salinity treatment due to handling error.

#### Wet mass change, percentage AFDM and physical condition

Salinity had a significant impact on the change in mean (±s.e.m.; *n*=10) net wet mass (low: −0.95±0.28 g; medium: −0.18±0.11 g; control: 0.021−0.13 g) (Welch's ANOVA: *F*_2_=4.90, *P*=0.021) (Table S1). There were significant differences between the low and control treatments (Games–Howell: *P*=0.019) (Table S1).

Salinity had a significant impact on mean AFDM as a percentage of total dry mass (Kruskal–Wallis: χ^2^_2_=6.86, *P*=0.03) (Table S1). The low salinity treatment had the lowest mean (±s.e.m.) percentage of AFDM to dry mass (low: 16.14±0.83%; medium: 19.5±1.13%; control: 18.11±0.65%); however, the difference was only significant between the low and medium salinity treatments (Dunn’s test: *P*=0.0046) (Table S1).

**Fig. 4.**
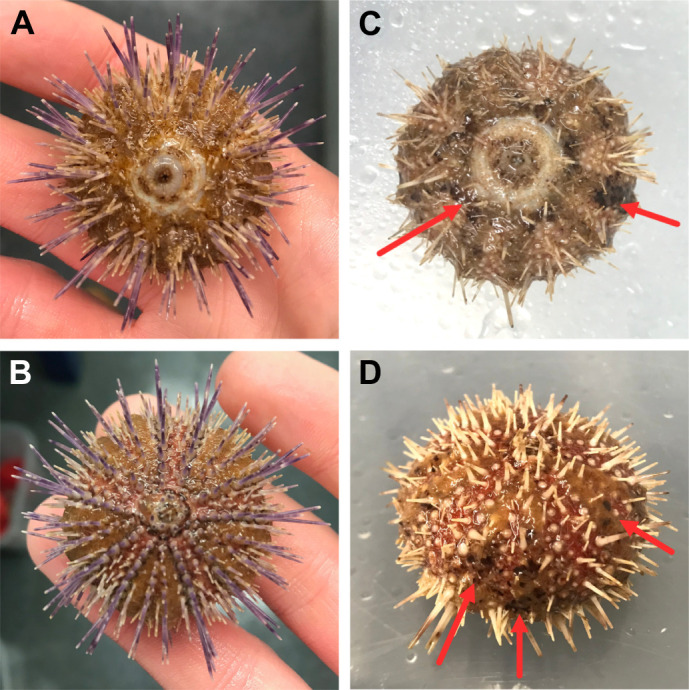
**Images of *E. esculentus* following exposure to ambient and low salinity treatment for 25 days.** (A,B) Oral and aboral view after 25 days in 31‰ (control treatment). (C,D) Oral and aboral view after 25 days in 21‰ salinity (low salinity treatment). Note the high primary spine loss and the tube foot damage (arrows) in the low salinity treatment.

At the end of the experimental period, urchins in the low salinity treatment had lost a high number of primary spines, which appeared to have detached at the tubercles, in addition to damaged tube feet, which were dark in colour (Fig. 4) and appeared limp when underwater. On visual inspection, the medium salinity urchins were indistinguishable from those in the control treatment.

#### Oxygen consumption

**Fig. 5.**
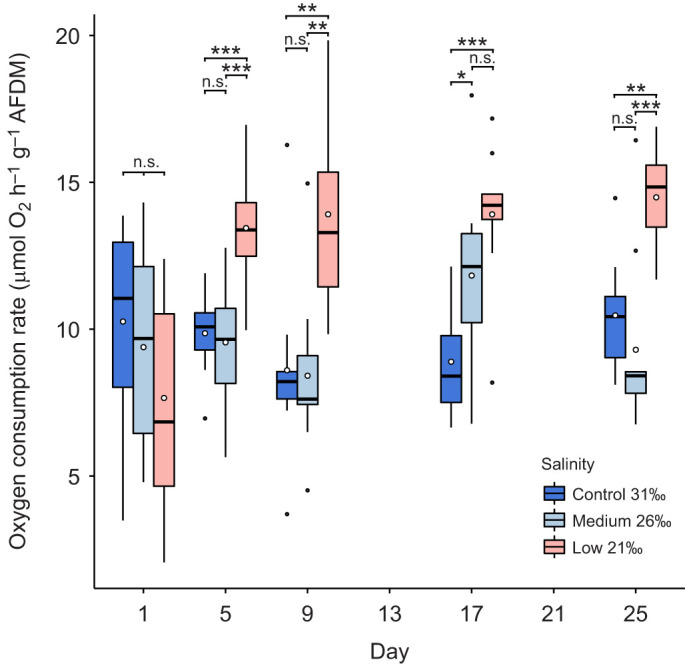
**Rate of oxygen consumption of *E. esculentus* at time points during the 25 day exposure to different salinity conditions.** Results are shown for one-way ANOVA and *post hoc* Tukey test between salinity treatments at each time point (n.s., not significant; *P<0.05, **P<0.01, ***P<0.001; *n*=10 biological replicates at each time point and treatment). Box plots show medians (black line), upper and lower quartiles, and maximum and minimum (whiskers). Means are represented by open circles and outliers by filled circles.

Rates of oxygen consumption varied significantly between salinity treatments (two-way ANOVA: *F*_2_=7.39, *P*=0.003) and time points (two-way ANOVA: *F*_4_=4.74, *P*=0.002), including their interaction (two-way ANOVA: *F*_8_=8.16, *P*<0.0001) (Fig. 5; Table S1). On day 1, oxygen consumption did not differ significantly between salinity treatments (ANOVA: *F*_2_=1.29, *P*=0.292). However, by day 5, oxygen consumption in the low salinity treatment was higher than that in both the medium and control treatments (Tukey: *P*<0.001) (Fig. 5; Table S1) and remained higher than the control for the duration of the experiment. The medium salinity treatment was not significantly different from the control except on day 17 (Tukey: *P*=0.042) (Fig. 5; Table S1).

#### Righting ability

**Fig. 6.**
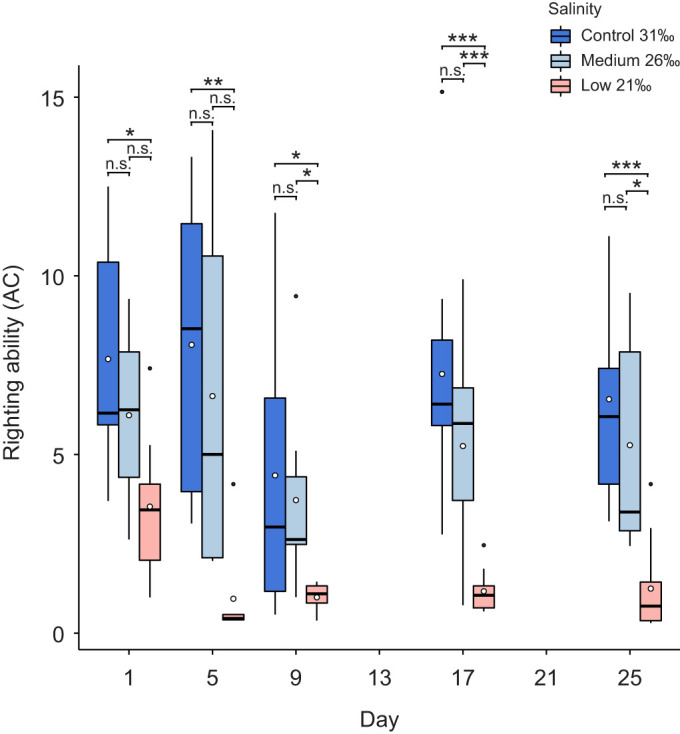
**Righting ability of *E. esculentus*** **at time points during the 25 day exposure to different salinity conditions.** Note: a smaller AC value indicates longer righting times. Results are shown for one-way ANOVA and *post hoc* Tukey test (days 5, 9 and 17) and Games–Howell test (days 1 and 25) between salinity treatments at each time point (n.s., not significant; *P<0.05, **P<0.01, ***P<0.001; *n*=7–9, 7–8, 7–10 biological replicates at each time point and treatment in control, medium and low salinity, respectively). Box plots show medians (black line), upper and lower quartiles, and maximum and minimum (whiskers). Means are represented by open circles and outliers by filled circles.

Mean AC values varied significantly between salinity treatments (two-way ANOVA: *F*_2_=69.23, *P*<0.0001) and time points (two-way ANOVA: *F*_4_=5.8, *P*<0.001), including their interaction (two-way ANOVA: *F*_8_=2.16, *P*=0.036) (Fig. 6; Table S1). The time taken for urchins to fully right themselves (expressed as AC values) was significantly longer in the low compared with the control salinity treatment at every sampling point throughout the experimental period (days 1, 9 and 17: Tukey, all *P*≤0.05; days 5 and 25: Games–Howell, *P*≤0.05) (Fig. 6; Table S1). AC values also differed significantly between the medium and low salinity treatment on days 9 and 17 (Tukey: *P*≤0.05) and day 25 (Games–Howell: *P*≤0.05). There was no significant difference in AC values between the medium and control treatments at any sampling point.

#### Feeding rate

**Fig. 7.**
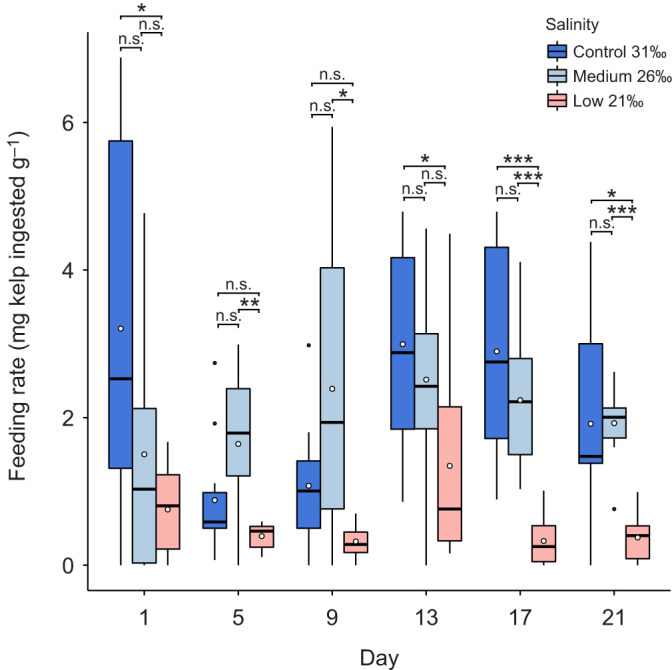
**Feeding rate of *E. esculentus*** **at time points during the 25 day exposure to different salinity conditions.** Feeding rate was calculated as the amount of kelp eaten in 24 h (buoyant wet mass per wet mass of urchin). Results are shown for one-way ANOVA and *post hoc* Tukey test (day 13) and Games–Howell test (days 1, 5, 9, 17 and 25) between salinity treatments at each time point (n.s., not significant; *P<0.05, **P<0.01, ***P<0.001; *n*=10 biological replicates at each time point and treatment). Box plots show medians (black line), upper and lower quartiles, and maximum and minimum (whiskers). Means are represented by open circles and outliers by filled circles.

Mean feeding rate varied significantly between salinity treatments (two-way ANOVA: *F*_2_=23.06, *P*<0.00001) and time points (two-way ANOVA: *F*_5_=6.32, *P*<0.0001), including their interaction (two-way ANOVA: *F*_10_=2.96, *P*=0.002) (Fig. 7; Table S1). At each feeding sample time point, the rate of feeding was significantly different between the control and low salinity treatment (Games–Howell: *P*<0.05) (Fig. 7; Table S1), except for days 5 and 9. The rate of feeding was also significantly different between the medium and low treatments for all feeding sample days (Games–Howell: all *P*<0.05) (Fig. 7; Table S1), except days 1 and 13. There were no significant differences in feeding rate between the control and medium treatment at any point in the experiment.

#### Coelomic fluid osmolality

**Fig. 8.**
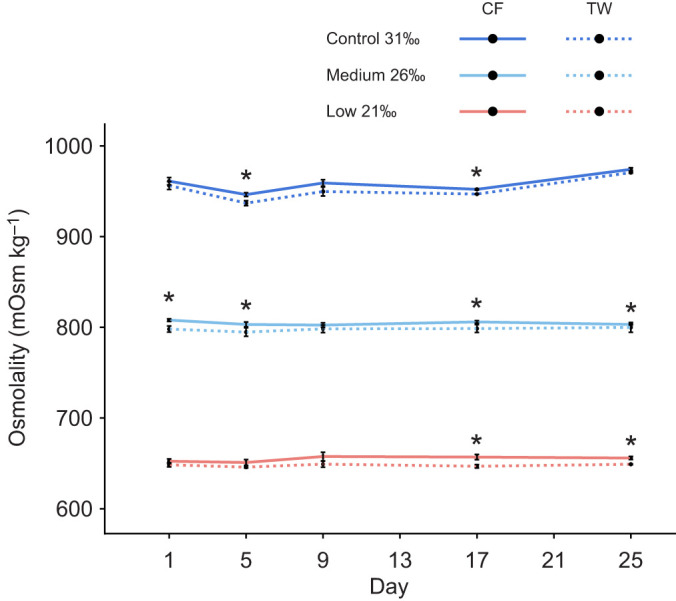
**Coelomic fluid osmolality of adult *E. esculentus* versus tank water osmolality.** Data are mean±s.e.m. coelomic fluid (CF; *n*=5 biological replicates for each salinity and time point) and tank water (TW; *n*=3 technical replicates for each salinity and time point) osmolality at time points spanning 25 days of exposure to different salinity conditions. Asterisk indicates a significant difference (one-sample *t*-test: *P*<0.05; *n*=5) between corresponding coelomic fluid and tank water values.

Mean coelomic fluid osmolality differed significantly between each treatment at every sample point, including their interaction (two-way ANOVA: *F*_8_=194, *P*≤0.0001; Tukey: all *P*<0.05) (Fig. 8; Table S1). Compared with the tank water, coelomic fluid was always slightly hyperosmotic at every sample point within each treatment, although this was only significant on days 5 and 17 in the control (all one-sample *t*-tests: day 5: *t*_4_=4.43, *P*=0.011; day 17: *t*_4_=7.74, *P*=0.001), days 1, 5, 17 and 25 in the medium salinity treatment (all one-sample *t*-tests: day 1: *t*_4_=6.88, *P*=0.002; day 5: *t*_4_=3.02, *P*=0.039; day 17: *t*_4_=5.25, *P*=0.006; day 25: *t*_4_=6.55, *P*=0.003) and days 17 and 25 in the low salinity treatment (all one-sample *t*-tests: day 17: *t*_4_=3.38 *P*=0.028; day 25: *t*_4_=3.94, *P*=0.015) (Fig. 8; Table S1).

#### Coelomocyte analysis

Mean total coelomocyte concentration after 25 days in experimental salinities showed no significant difference between treatments (Table 1). The percentage of viable cells and red spherulocytes as a proportion of total coelomocytes also showed no significant difference between treatments (Table 1).

**Table 1.**
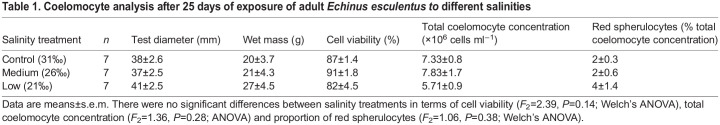
Coelomocyte analysis after 25 days of exposure of adult *Echinus esculentus to* different salinities

### Experiment 3: hypo-osmotic shock of acclimated urchins

**Fig. 9.**
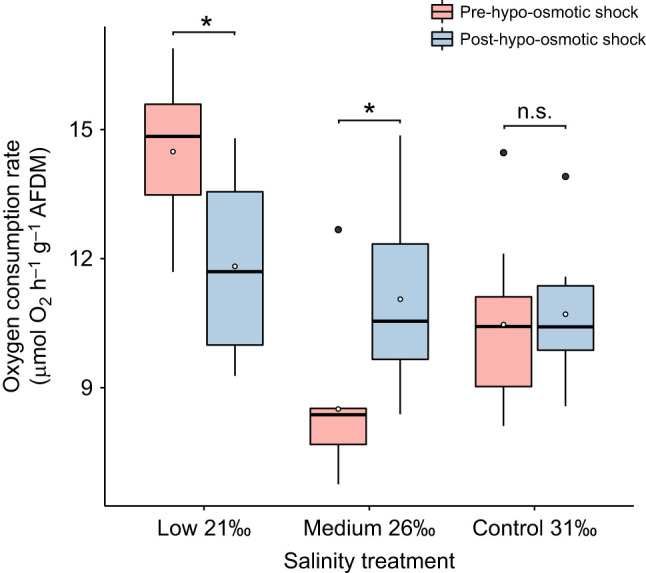
**Oxygen consumption of *E. esculentus* before and after hypo-osmotic shock following acclimation to reduced salinity.** *Echinus esculentus* were exposed to different salinity treatments for 25 days (red boxes: *n*=10 biological replicates) followed by an acute immersion in 18‰ salinity (hypo-osmotic shock; blue boxes: *n*=8 biological replicates). Asterisk indicates a significant difference (two-sample *t*-test: *P*<0.05) between mean oxygen consumption pre- and post-acute immersion for each salinity treatment (n.s., not significant). Box plots show medians (black line), upper and lower quartiles, and maximum and minimum (whiskers). Means are represented by open circles and outliers by filled circles.

Following acclimation, mean oxygen consumption rate after acute immersion in 18‰ salinity showed similar values between acclimated treatments and there were no significant differences (Fig. 9; Table S1). However, oxygen consumption within the medium salinity treatment increased significantly after acute immersion when compared with its prior acclimated value, while that for the low treatment conversely decreased significantly (two-sample *t*-test: medium, *t*_15_=−2.74, *P*=0.015; low, *t*_16_=2.86, *P*=0.011) (Fig. 9; Table S1), indicating a very different response. The control treatment showed no significant change after acute immersion.

After 5 h in 18‰ salinity, two urchins from the medium salinity treatment righted fully within 30 min (at 17 and 29 min). None of the urchins in the low or control salinity treatments righted within 1 h or showed any signs of righting (i.e. tube feet remained limp).

Mean coelomic fluid osmolality varied significantly between treatments after 6 h in 18‰ salinity (Welch's ANOVA: *F*_2_=15.82, *P*=0.0036), with that of the low and medium salinity treatments being significantly lower than the control (Games–Howell: low versus control, *P*=0.008; medium versus control, *P*=0.034) (Tables S1 and S3). The coelomic fluid osmolality in all treatments was significantly higher than the tank water osmolality (one-sample *t*-test: control, *t*_4_=7.97, *P*=0.001; medium, *t*_4_=8.04, *P*=0.001; low, *t*_4_=25.96, *P*<0.0001) (Table S1).

After 72 h in the recovery tanks, all animals had survived. Righting ability was assessed (*n*=8 per treatment) and all animals righted fully within 13 min.

## DISCUSSION

Salinity acclimation is distinct from short-term salinity tolerance, as the former involves a process of cellular reorganisation that aids in long-term survival within the altered environment, while the latter constitutes a temporary ‘reflex’ response to osmotic stress (Khlebovich, 2017; Rivera-Ingraham and Lignot, 2017). This study revealed that *E. esculentus* has a 24 h lower tolerance limit of 21‰ after direct transfer from ambient conditions (with a predicted LC_50_ threshold of 18.5‰), characterised by metabolic depression and a decline in activity. In contrast, exposure to a salinity of 21‰ over 25 days led to an increase in metabolic rate, while activity and feeding were impaired. This resulted in a decline in physical condition, suggesting that this falls below the plasticity limit of acclimation. At 26‰ salinity, there were no measurable impacts on urchin behaviour or physiology after 25 days of exposure, indicating successful acclimation. The hypo-osmotic shock trial demonstrated that acclimation offers beneficial functional resistance (righting ability and metabolic capacity) upon exposure to further osmotic challenges.

### Survival and physical condition

Acute low salinity tolerance thresholds in echinoids are species and population specific, often reflecting natural distributions (Russell, 2013; Stickle and Diehl, 1987). The lower survival limit of 21‰ established for *E. esculentus* probably reflects their subtidal preferred habitat (Comely and Ansell, 1988). Indeed, LC_50_ salinity data from intertidal populations of *Psammechinus miliaris* (collected from the same area as *E. esculentus*) were observed to be substantially lower (<16‰; N.J.B., unpublished results; see also Gezelius, 1963), suggesting that salinity is a significant factor in an echinoid’s depth distribution.

Although there was no significant impact on survival in the acclimation experiment, there was an observable decline in the physical condition of urchins at 21‰. The high spine loss and appearance of dark coloration in the tube feet indicated severe stress. Significantly, the reduction in mass recorded at the end of the acclimation experiment suggests a cessation of growth and tissue breakdown. If the cost of osmoregulation is too high, energy allocation for processes such as growth and maintenance may be limited, and when severe, tissue may be catabolised to sustain energetic demands (Podbielski et al., 2022). The difference in the ratio of AFDM to dry mass between the low and higher salinity treatments suggests salinity had an impact on organic mass despite high spine loss (although spine loss was not quantified), indicating depletion of stored reserves and possibly tissue catabolism. Reductions in mass have previously been observed in echinoids including *P. miliaris* and *Strongylocentrotus droebachiensis* held in low salinities over 6 weeks (Podbielski et al., 2022). Spine loss has also been observed in urchins held in long-term low salinity experiments (Sabourin and Stickle, 1981). Physical damage as a result of abiotic stress has been implicated in the reduced survival rates and ability of urchins to recover when ambient conditions return to normal (Minuti et al., 2021). Despite survival at 21‰ over the experimental period, the declines in physical condition observed may have detrimental impacts on urchin recovery ability and ultimately long-term survival.

### Physiological functions

Oxygen consumption varied over the time frame of exposure and with the degree of salinity reduction. Previous echinoderm studies investigating the effects of acute low salinity exposure on oxygen consumption have showed varied responses (Stickle and Diehl, 1987). Reductions in oxygen consumption have been observed in echinoids, including *Strongylocentrotus purpuratus* (Giese and Farmanfarmaian, 1963) and *S. droebachiensis* (Sabourin and Stickle, 1981). Similar reductions have been observed in an asteroid (Shirley and Stickle, 1982b) an ophiuroid (Talbot and Lawrence, 2002) and a holothurian (Sabourin and Stickle, 1981). Conversely, Stickle and Diehl (1987) reported an inverse relationship between oxygen consumption and salinity between 30‰ and 15‰ in the ophiuroid *Ophioderma brevispinum*. In the current study, the initial metabolic depression observed under acute low salinity (below 26‰) indicated a reduction in energy utilisation. It is unlikely that this is a result of animals dying (survival was 100% at 21‰ and above) and more likely to be a consequence of the rapid influx of water into tissue via osmosis, leading to a temporary slowdown in physiological activity. Metabolic rate depression can be a reaction to extreme stress where an organism reduces activity and energy consumption, prioritising energy allocation in order to maintain homeostasis (Sokolova et al., 2012). However, it is important to note the current study only recorded the initial metabolic response to a hypo-osmotic exposure (hours 2–5 out of 24), potentially missing a change in the metabolic response once a critical threshold was breached. At 26‰, the absence of metabolic depression indicated sufficient energy was available to facilitate a relatively quick response to the change in salinity. This may reflect the higher energy demand for osmoregulatory cellular processes entrained to counter the acute osmotic stress (Rivera-Ingraham and Lignot, 2017).

Longer term studies have demonstrated a reduction in oxygen consumption after acclimation to reduced salinity in echinoderms. For example, oxygen consumption in *S. droebachiensis* after 14 days in low salinity (15‰) was lower than at ambient (30‰) in the summer (Sabourin and Stickle, 1981). A similar response was observed in the asteroid *Leptasterias hexactis*, which exhibited significantly lower oxygen consumption after 28 days of exposure to lowered salinity (15‰ and 20‰) than at ambient (30‰) (Shirley and Stickle, 1982b). In the current study, the higher rates of oxygen consumption after chronic exposure to low salinity diverge from the results seen in previous studies. The impact of the postprandial rise in metabolism can be excluded as a cause because feeding in the low salinity urchins had largely ceased. For *E. esculentus*, therefore, the results suggest that increased energy expenditure was needed to account for costs related to the acclimation process (Sokolova et al., 2012). These costs may be attributed to cellular rebalancing processes, such as modification of membrane-bound transporters and deamination of amino acids to reduce the cellular osmolyte pool (Gilles, 1987; Gilles and Delpire, 1997; Podbielski et al., 2022).

Under chronic low salinity (21‰), the increase in oxygen consumption did not correlate with an increased righting response. Righting times were significantly longer compared with those for control and medium salinities, and did not improve throughout the experimental period. The acute response data showed significant reductions in righting ability below 26‰, suggesting both acute and chronic low salinity disrupt neuro-muscular coordination in *E. esculentus*, perhaps due to tissue swelling and the inability to restore cellular volume. Previous studies have reported a diminished righting ability in echinoids exposed to lowered salinity [e.g. ﻿*S. droebachiensis* (Moura et al., 2023; Sabourin and Stickle, 1981) and *Lytechinus variegatus* (Lawrence, 1975)], a finding supported by this study. The righting response has often been described as a measure of ‘functional health’, with studies demonstrating that it is positively correlated with the energy budget as a function of salinity in echinoderms (Stickle and Diehl, 1987). The decline in righting performance under chronic low salinity, despite the increase in oxygen uptake, implies a limitation in the capacity to utilise oxygen efficiently. This suggests that salinity is reaching a limiting value, perhaps analogous to the oxygen- and capacity-limited thermal tolerance concept, which proposes that an animal is constrained by physiological limitations in oxygen delivery under thermal stress (see Pörtner et al., 2017).

Crucially, high metabolism must be matched by high food intake or a switch to stored energy reserves. In low salinity, feeding was dramatically lower than at ambient and remained consistently low throughout the experimental period, indicating severe stress. Previous studies have demonstrated reduced feeding rates in echinoderms exposed to low salinity [for example: *L. hexactis* (Shirley and Stickle, 1982a), *Pisaster ochraceus* (Held and Harley, 2009)]. Held and Harley (2009) hypothesised that the reduction in activity under salinity stress supresses the physical ability to feed in asteroids. This is supported by the current study, which shows a reduction in righting ability combined with reduced feeding.

### Impact of salinity on coelomic fluids

Throughout the acclimation experiment, coelomic fluid osmolality values were slightly hyperosmotic to the seawater at each salinity (including ambient), indicating a small degree of ionic regulation, consistent with findings in other echinoids such as *﻿L. variegatus* (Vidolin et al., 2007). Previous studies have reported that echinoid coelomic fluid is generally isosmotic or slightly hyperosmotic compared with seawater (Diehl, 1986; Santos et al., 2013; Vidolin et al., 2007). Robertson (1980) observed near-identical osmolality values between the coelomic fluid from *E. esculentus* and seawater measured at ambient salinity, similar to the current study. With acute exposure, coelomic fluid osmolality was similar to corresponding levels observed in the acclimation study, suggesting that full osmotic rebalancing of the extracellular fluid is complete within 24 h. Interestingly, coelomic fluid taken from urchins after 24 h in 16‰ and 11‰ salinity had larger osmotic gradients (i.e. the difference in osmolality between the coelomic fluid and the seawater) than those at 21‰ and above, even though the urchins were probably dying or dead. This warrants further investigation, perhaps through measuring the degree of tissue hydration after immersion in low salinity.

The hypo-osmotic shock trial on the acclimated urchins showed that coelomic fluid osmotic gradients in all treatments were substantially larger at 6 h than at 24 h, indicating that extracellular rebalancing was incomplete and that significant gradients were maintained. Numerous echinoid species have demonstrated positive ionic and osmotic gradients between the coelomic fluid and seawater after immersion in low salinity, which persist for a number of hours (reportedly 6–12 h) (Diehl, 1986; Freire et al., 2011; Vidolin et al., 2007). Despite being considered a subtidal urchin, *E. esculentus* maintains a hyperosmotic gradient for several hours after low salinity immersion. This ability may protect body tissues from sudden extreme hypo-osmotic shock, akin to intertidal species (Freire et al., 2011; Stickle and Ahokas, 1974; Vidolin et al., 2007).

Long-term exposure to low salinity appears to have no demonstrable impact on coelomocyte viability, total cell abundance or percentage of red spherulocytes. Abiotic stressors have previously been shown to affect coelomocyte composition, with significant increases of red spherulocytes after acute heat stress (Branco et al., 2012). Furthermore, a study examining the impact of acute hypo-osmotic stress on coelomocytes in the urchin *Echinometra lucunter* observed a 122.5% increase in total coelomocyte abundance after 24 h, but no change in red spherulocyte number (Honorato et al., 2017). In the current study, only chronic exposure to low salinity was examined; therefore, the impact of acute low salinity stress on the immune cells of *E. esculentus* remains unknown.

### Acclimation at 26‰ salinity

Acclimation is commonly defined as the stabilisation of physiological functions after a change in conditions that are experimentally altered (Prosser, 1991; Schmidt-Nielsen, 1997). By the end of the experimental period, measures of oxygen consumption, feeding and activity in urchins exposed to 26‰ were similar to those at ambient salinity. Furthermore, there were no significant effects on growth or visible impacts on physical condition. This suggests that acclimation had been achieved in the medium treatment by the end of the experiment, confirming part of the original hypothesis (hypothesis 2). Additionally, it was predicted that acclimating to reduced salinity would confer functional resistance to subsequent acute hypo-osmotic immersion. Following acclimation and immersion in 18‰, righting ability was still functional in a small number of individuals in the medium treatment, while absent in both the control and low treatment. Furthermore, the metabolic response to the osmotic shock varied between the low and medium treatments, with a higher metabolic rate observed in the medium treatment. An increase in metabolic rate indicates sufficient energetic resources are available, offering an explanation for the ability of some urchins to right. The osmolality data from the hypo-osmotic shock trial, showed that the osmotic gradient was greatest in the control treatment (Table S3), suggesting that intracellular osmotic pressure was higher than for the medium treatment. The larger gradient would cause an increase in cell volume, resulting in tissue swelling. This appears to have had a direct impact on physiological functioning, as evidenced by the inability of the control treatment to produce a righting response.

Overall, long-term exposure to 26‰ appears to have given a beneficial advantage to *E. esculentus* in the face of further hypo-osmotic exposure and offers further evidence that acclimation to 26‰ was achieved. Successful acclimation to mild stressors (e.g. a small rise in temperature or small reduction in pH) has been previously shown to have beneficial impacts in echinoids, such as improving reproductive outcome (Suckling et al., 2015). However, further investigations are necessary to establish whether costs related to osmotic acclimation in *E. esculentus* are traded off with investment in reproduction or longer term somatic growth as demonstrated in other echinoids and osmoconformers (Sanders et al., 2018; Santos et al., 2022).

### Lower limit of salinity acclimation

Unlike in the medium treatment, stabilisation of physiological functions did not occur in the low salinity urchins. Instead, metabolic rate was substantially elevated to meet the cellular osmoregulatory demands. The increased metabolic costs were clearly not met through food intake. Feeding rates were significantly impacted in the lowest salinity, thereby creating an energetic deficit explaining the mass reduction and possible catabolic tissue breakdown. Furthermore, the resulting damage to the tube feet would further restrict locomotion and feeding, while also reducing the surface area available for respiration (Hyman, 1955; Leddy and Johnson, 2000; Moura et al., 2023). Without a shift towards a positive energy balance through feeding, the metabolic deficit would continue to increase cumulatively, eventually resulting in a decline in urchin health and/or mortality. At the cellular level, the osmolality data from the low salinity treatment confirms that extracellular coelomic fluids were consistently isosmotic or slightly hyperosmotic with the external salinity. Previous studies show that full cellular ionic rebalancing can be achieved within 14 days in many marine invertebrates (e.g. *Geukensi demissa*; Baginski and Pierce, 1977), and is therefore also likely here. Under lowered salinity, echinoids reduce their intracellular organic osmolyte pool, which primarily consists of the amino acid glycine (Podbielski et al., 2022). Therefore, the reduction of available essential amino acids may impact the ability of cells to perform other anabolic processes, such as protein synthesis, and may have contributed to the decline of physiological functioning as observed here under low salinity. The physiological and behavioural data demonstrate that urchins were not able to acclimate to the lowest salinity (21‰), indicating that the lower limit for acclimation is between 26‰ and 21‰.

### Ecological context

The urchins used in this study originated in Loch Linnhe. Here, estuarine circulations are created when high freshwater input, fed by multiple river systems and terrestrial run-off, mixes with incoming saltwater (Rabe and Hindson, 2017). This results in significant stratification, with fresher surface layers flowing seaward, and deeper, saltier layers flowing into the loch (Rabe and Hindson, 2017). Data from Upper Loch Linnhe show salinity is most variable in the top ∼5 m in spring, but by autumn when rainfall is high, strong salinity gradients are present throughout the top 20 m (e.g. minimum surface salinity was 28‰ and 31‰ in May, and 15‰ and 23‰ in October 2011 and 2012, respectively) (Rabe and Hindson, 2017). With knowledge of the salinity tolerance data from the current study and the salinity gradients recorded in Loch Linnhe, it is perhaps unsurprising that *E. esculentus* is known to favour depths below 15 m (Comely and Ansell, 1988). This suggests that salinity probably plays a significant role in shaping *E. esculentus* distribution patterns. Therefore, shallow lochs fed by high freshwater inputs are unlikely to be favourable to *E. esculentus* at present and in the future, especially if the rate of extreme freshwater events increases in the UK as a result of changing weather patterns (Adaptation Scotland, www.adaptationscotland.org.uk/why-adapt/climate-trends-and-projections, accessed 29 August 2023). A change in *E. esculentus* distribution may have a cascading effect on the numerous macroalgal species upon which they graze, as previously observed (Bekkby et al., 2015; Jones and Kain, 1967).

### Future directions and conclusions

The *E. esculentus* urchins used in this study represent smaller and younger adults, as older individuals can have test diameters of 150–160 mm (Mortensen, 1927). Echinoid body size is certainly relevant in relation to tolerating reduced salinity (Castellano et al., 2017); however, previous authors have recognised that size is not the only factor defining salinity tolerance and buffering capabilities in echinoderms (Freire et al., 2011; Santos et al., 2013; Vidolin et al., 2007). Indeed, Stickle and Denoux (1976) note that smaller specimens of *S. droebachiensis* were more tolerant of low salinity than larger specimens, while Vidolin et al. (2007) demonstrated that the osmotic and ionic gradients established under reduced salinity were independent of surface area to volume ratio in *L. variegatus.* However, considering the very large size of fully grown *E. esculentus*, it would be of interest to establish how size influences its salinity tolerance.

Controlled salinity acclimation experiments differ from the natural acclimatisation process that *E. esculentus* may exhibit in the wild. Salinity fluctuates on a daily time scale as a result of tidal changes and weather patterns, while longer term reductions in salinity as a response to changing weather patterns will probably be gradual. What acclimation experiments do offer is a prediction of how *E. esculentus* may respond to salinity stress *in situ*; however, the current study is unable to determine the long-term success of *E. esculentus* under repeated periods of salinity stress. The impact of a period of extreme stress can be severely detrimental to the long-term health of urchin populations, as vital energy resources can be diverted away from reproduction and growth (Santos et al., 2022). Conversely, successful acclimation to mild stressors can prove beneficial for reproductive success and larval outcomes in echinoids (as shown by Suckling et al., 2015). Furthermore, early life stages are considered more sensitive to environmental stressors than in adult urchins (Bressan et al., 1995). Indeed, even small reductions in salinity have proved to be highly damaging to urchin embryos (Cowart et al., 2009). Future research on the impact of periodical low salinity stress on reproduction and growth in *E. esculentus* and other echinoderms would now be valuable. Studies investigating low salinity tolerance and acclimation across a full life cycle are needed to allow predictions of future resilience to change.

The mechanisms underpinning why some echinoderm species have greater euryhaline tolerance and acclimation ability than others are still largely unknown. Progress has been made with metabolomic approaches in identifying osmolytes used in cellular osmoregulation as part of the acclimation process (Podbielski et al., 2022). However, there appears to be no transcriptomic analysis for echinoderms in the context of osmotic stress. This is surprising as such studies exist for many other marine invertebrates for identifying genetic responses to osmotic stress (e.g. Meng et al., 2013; Lou et al., 2019; Barrett et al., 2022). A more recent development in environmental research is the integration of omics techniques to offer a multi-omics approach (e.g. Ichihashi et al., 2020; Bakker et al., 2023). This has the potential to identify causal links between physiological fitness parameters and their underlying genetic and biochemical signatures and would offer a holistic understanding on the nature of osmotic acclimation and tolerance in echinoderms.

In summary, the current study has demonstrated that adult *E. esculentus* can acclimate to lowered salinity with beneficial consequences in terms of further hypo-osmotic challenges. However, at the lower threshold of the acute tolerance range (21‰), chronic exposure has severe detrimental impacts on physiological functioning and activity, although innate immune cell composition and concentration were unaffected. Therefore, 21‰ salinity is probably beyond the phenotypic plasticity limit of *E. esculentu*s, and continued exposure may lead to mortality. For *E. esculentus* inhabiting coastal areas prone to freshening, such resilience will probably enable long-term survival at salinity levels of around 26‰; however, lower salinities are likely to represent a barrier to their distribution and survival in the face of extreme climate change, which, in turn, may also impact their macroalgae food source.

## Supplementary Material

10.1242/jexbio.246707_sup1 Supplementary information

Table S1.

## ECR Spotlight

This article has an associated ECR Spotlight interview with Nicholas Barrett.
