# Supplementary material for: Behavioural and physiological impacts of low salinity on the sea urchin Echinus esculentus
Source: J Exp Biol. 2024 Jan 16;227(2):jeb246707. doi: 10.1242/jeb.246707 (PMC10906488; doi:10.1242/jeb.246707)
Supplement: Supplementary information [file jexbio-227-246707-s1.pdf]

Table S1.

Available for download at
https://journals.biologists.com/jeb/article-lookup/doi/10.1242/jeb.246707#supplementary-data

Table S2. Tank effect analysis statistics.

Feeding	Kelp~Tank*Timepoint+Error(Animal/Timepoint)	Variables	d.f.	Mean sq	*F*	*P* value
	Control	Tank	2	4.16	0.979	0.4220
		Error: Animal	7	4.25	n/a	n/a
		Timepoint	5	10.43	5.436	0.0008
		Tank:Timepoint	10	2.72	1.421	0.2121
		Error: Animal:Timepoint	35	1.92	n/a	n/a
	Medium	Tank	2	0.66	0.140	0.8710
		Error: Animal	7	4.69	n/a	n/a
		Timepoint	5	1.69	1.239	0.3120
		Tank:Timepoint	10	2.11	1.549	0.1640
		Error: Animal:Timepoint	35	1.36	n/a	n/a
	Low (squared data)	Tank	2	0.10	0.414	0.6760
		Error: Animal	7	0.25	n/a	n/a
		Timepoint	5	0.40	3.986	0.0058
		Tank:Timepoint	10	0.17	1.691	0.1221
		Error: Animal:Timepoint	35	0.10	n/a	n/a
**Oxygen consumption**	**O2~Tank*Timepoint+Error(Animal/Timepoint)**	**Variables**	**d.f.**	**Mean sq**	***F***	***P* value**
	Control	Tank	2	0.53	0.027	0.9740
		Error: Animal	7	19.61	n/a	n/a
		Timepoint	4	6.89	1.716	0.1740
		Tank:Timepoint	8	3.42	0.853	0.5660
		Error: Animal:Timepoint	28	4.02	n/a	n/a
	Medium	Tank	2	5.83	0.191	0.8300
		Error: Animal	7	30.55	n/a	n/a
		Timepoint	4	26.16	2.674	0.0526
		Tank:Timepoint	8	4.63	0.473	0.8647
		Error: Animal:Timepoint	28	9.79	n/a	n/a
	Low	Tank	2	7.07	0.402	0.6840
		Error: Animal	7	17.59	n/a	n/a
		Timepoint	4	98.47	16.222	0.0000
		Tank:Timepoint	8	7.08	1.167	0.3530
		Error: Animal:Timepoint	28	6.07	n/a	n/a
**Righting (Activity coefficient)**	**AC~Tank*Timepoint**	**Variables**	**d.f.**	**Mean sq**	***F***	***P* value**
	Control	Tank	3	5.23	0.350	0.7900
		Timepoint	4	18.16	1.214	0.3300
		Tank:Timepoint	8	7.86	0.525	0.8260
		Error	25	14.96	n/a	n/a
	Medium	Tank	2	10.00	0.718	0.4990
		Timepoint	4	9.57	0.687	0.6090
		Tank:Timepoint	8	3.91	0.281	0.9650
		Error	22	13.92	n/a	n/a
	Low (Logged data)	Tank	2	0.50	0.917	0.4098
		Timepoint	4	3.30	6.031	0.0010
		Tank:Timepoint	8	0.44	0.802	0.6056
		Error	32	0.55	n/a	n/a

Table S3. Coelomic fluid osmolality (mean±s.e.m., *n*=6 biological replicates) at the end of the hypoosmotic shock trial (6 hours in 18‰ salinity water) that was conducted on the acclimated urchins on day 25.

**Treatment**	**Mean osmolality (±s.e.m mOsm kg^{-1})**	**Different letter indicates significant differences between groups**
Control (31‰)	631 ± 10	A
Medium (26‰)	595 ± 6	B
Low (21‰)	579 ± 1	B
Tank water (18‰)	552	

Journal of Experimental Biology • Supplementary information
